# Concentration quenching inhibition and fluorescence enhancement in Eu^3+^-doped molybdate red phosphors with two-phase mixing†

**DOI:** 10.1039/d3ra05873e

**Published:** 2023-11-01

**Authors:** Shuanglai Liu, Yimin Yan, Xiaohan Liu, Zheqian Cui, Shiheng Jia, Yiwen Xing, Shuang Guo, Bao Wang, Yunfeng Wang

**Affiliations:** a School of Physics and Electronic Engineering, Zhengzhou University of Light Industry Zhengzhou 450000 P. R. China; b School of Information Engineering, Nanyang Institute of Technology Nanyang 473004 P. R. China wangyunfeng@nyist.edu.cn

## Abstract

Red phosphor plays a crucial role in improving the quality of white light illumination and backlight displays. However, significant challenges remain to enhance red emission intensity in different matrix materials. Herein, a class of two-phase mixing red phosphors of NaIn_1−*x*_(MoO_4_)_2_:*x*Eu^3+^ (NIMO:*x*Eu^3+^) has been successfully prepared by the traditional high-temperature solid-state reaction method. The coordination environment, phase structure, excitation and emission spectra, fluorescence kinetics, and temperature-dependent luminescence properties of the system have been studied comprehensively. It is worth mentioning that the red emission intensity continues to increase with the increased Eu^3+^ doping concentration, and the fluorescence lifetimes remain unchanged. These extraordinary phenomena mainly stem from the special concentration quenching mechanism in such two-phase mixing material, namely, the increased lattice interface barriers from Eu six-coordinated units and Eu eight-coordinated units can effectively block the non-radiation by enlarging the average distance between luminescent centers. The improved fluorescence thermal stability and suppressed non-radiative transition rate in NIMO:40%Eu^3+^ sample are further proving regulatory role of lattice interface barriers. In addition, a warm white light-emitting diode (LED) is successfully fabricated, exhibiting Commission Internationale de l'Eclairage (CIE) coordinates of (0.343, 0.335), a color rendering index (CRI) of 92.1, and a correlated color temperature (CCT) of 5013 K, showing significant application prospects for high-quality lighting devices.

## Abstract 2

Due to the regulatory effect of lattice interface barriers, the two-phase mixing NaIn(MoO_4_)_2_:Eu^3+^ phosphors exhibit bright red light emission and concentration quenching inhibition behavior.

## Graphical abstract

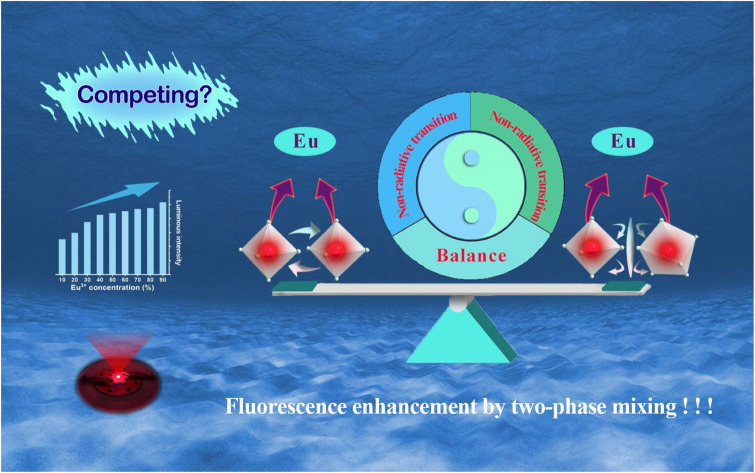

## 1. Introduction

In recent years, inorganic luminescent materials have received increasing attention from scholars due to their widespread applications in lighting, displays, optical communication, solar cells, biological detection, and other fields.^1–7^ Currently, a popular lighting solution for producing white light is the utilization of commercial blue fluorescent LED chips combined with yellow light YAG:Ce^3+^ phosphors.^8–10^ However, the problems of high color temperature and low color rendering index need to be further solved in the warm white lighting field due to the lack of red components.^11^ With further research, Mn^4+^ doped fluoride red light material as an excellent candidate is gradually being utilized in the production of white LEDs to make up the shortcoming, but poor moisture resistance is the key factor restricting its promotion and application.^12,13^ Therefore, it is still necessary to explore alternative stable and efficient red light materials for warm white LEDs.

Eu^3+^ ions, with a typical 4f–4f electronic layer transition, are minimally influenced by external electromagnetic fields and demonstrate highly efficient near-ultraviolet excitation and conversion into red emission.^14^ In recent years, Eu^3+^ activated oxide red light materials play an important role in the production of near-ultraviolet (NUV) excited white LED devices, because of its excellent physicochemical stability and favorable luminescent performance. Eu^3+^ doped red phosphors have been extensively studied, such as in silicate, borate, vanadate, phosphate, molybdate, tungstate, organic complexes and other systems.^15–20^ Considering the luminous efficiency, the number of effective Eu^3+^ centers is one of the important influencing factors. In general, the cross-energy transfer (CET) among Eu^3+^ ions increases becomes more serious as the luminescent centers increases (doping concentration increases), leading to the occurrence of concentration quenching effects. Many studies have clarified that the CET relation can be effectively changed through the regulation of micro-structure, such as in La_2_W_2_O_9_:Eu^3+^, Ca(Sr)Al_12_O_19_:Eu^3+^, MBaY(BO_3_)_2_:Eu^3+^ (M = Na, K), KLa_1−*x*−*y*_(MoO_4_)_2−*z*_(WO_4_)_*z*_:*x*Eu^3+^/*y*Dy^3+^, CaMoO_4_:Eu^3+^/Na^+^.^21–25^ In these numerous methods, entering a reasonable crystal field distortion by replacing non-rare-earth ions with rare-earth luminescent centers is an effective approach for improving luminescence performance. For LaSc_3_(BO_3_)_4_:Eu^3+^ red light material, the host lattice gradually shifts from phase α to β with the increasing Eu^3+^ concentration. At the same time, due to the increasing luminescence center spacing, there is a significant improvement in luminescence efficiency, while also exhibiting excellent luminescent thermal stability.^26,27^ In certain materials, there exists a notable separation between the positions occupied by luminescence centers. This spatial disparity becomes evident in instances such as the extended distance between emission centers in phosphors like Li_3_BaSrLa_3_(MoO_4_)_8_:Eu^3+^ and LiCaLa(MoO_4_)_3_:Eu^3+^. It is crucial to underscore that this spatial separation plays a pivotal role in influencing both emission efficiency and thermal stability. The discernible impact on these properties emphasizes the intricate relationship between the arrangement of luminescence centers and the overall performance characteristics of the aforementioned phosphors.^28,29^ Our recent study has found that in the non-equivalent substituted SrWO_4_:Eu^3+^ phosphor, a second phase structure of Eu_2_W_2_O_9_ appears as the Eu^3+^ concentration increases and an associated luminescence enhancement presents when the Eu^3+^ concentration reaches a certain level.^30^ Therefore, further exploration is needed to improve the luminescence efficiency and stability of red light materials by using the strategies of changing host structure.

In this work, a series of NIMO:*x*Eu^3+^ (*x* = 10–90%) red fluorescent powders are prepared using a high-temperature solid-state reaction method. Accompanied by significant differences in atomic radius between Eu^3+^ and In^3+^ ions, two crystal structures of Eu^3+^, six-coordinated and eight-coordinated, are formed in host material. The spectral analysis indicates that the typical luminescence concentration quenching phenomenon of Eu^3+^ is suppressed while obtaining enhanced luminescence. A novel concentration quenching mechanism on regulating the non-radiative transition rate in two-phase mixing material is suggested to explain the enhancement of luminescence, invariant fluorescence lifetimes, and improved luminescence thermal stability. Moreover, the prepared white LED exhibits superior luminous performance on CIE of (0.343, 0.335), CRI of 92.1 and CCT of 5013 K, which can meet the requirements of high-quality lighting applications.

## 2. Experimental section

### 2.1 Sample preparation

The raw materials, including Na_2_CO_3_ (99.99%), In_2_O_3_ (99.99%), MoO_3_ (99.99%), and Eu_2_O_3_ (99.99%), are used to prepare NIMO:*x*Eu^3+^ phosphors through a high-temperature solid-state method. Chemical ratios dictate the precise weight of each raw material used, and samples with varying doping ratios (*x* = 10–90%) are prepared. These samples are then finely ground in a mortar for 30 minutes before being placed in an alumina corundum crucible. The mixture is heated at a rate of 3 K min^−1^ in a muffle furnace to 923 K and calcined for 8 hours. Following cooling to room temperature, the samples are retrieved for grinding and subsequent qualitative and quantitative analysis. The preparation of pc-LED is achieved by mixing a certain proportion (1 : 1) of phosphors with UV curing adhesive, and then coating it on a NUV LED chip.

### 2.2 Sample characterization

A CuK α radiation source was used with an X-ray diffractometer (XRD), at a tube current of 40 mA and tube voltage of 40 kV, to analyze the phase structure of samples. The Beijing Zhuolihanguang Instrument Co., Ltd provided the OmniFluo900 fluorescence spectrometer to conduct excitation, emission, and fluorescence lifetime tests on the samples using a 75 W xenon lamp and 20 Hz pulse laser. The fluorescence spectrometer was combined with the Oxford Low Temperature Opto-electronic Test and Analysis System to investigate the thermal stability index of the sample, with a sampling interval of 25 K in a variable temperature environment of 100–500 K. In addition, absolute quantum yield measurements were conducted using the Edinburgh Instruments FS5 fluorescence spectrometer equipped with an attached integrating sphere module. Electroluminescence spectra of the pc-LEDs were performed by the Auto-Temperature LED Opto-electronic Analyzer of EVERFINE.

## 3. Results and discussion

### 3.1 Crystal structure analysis

**Fig. 1:**
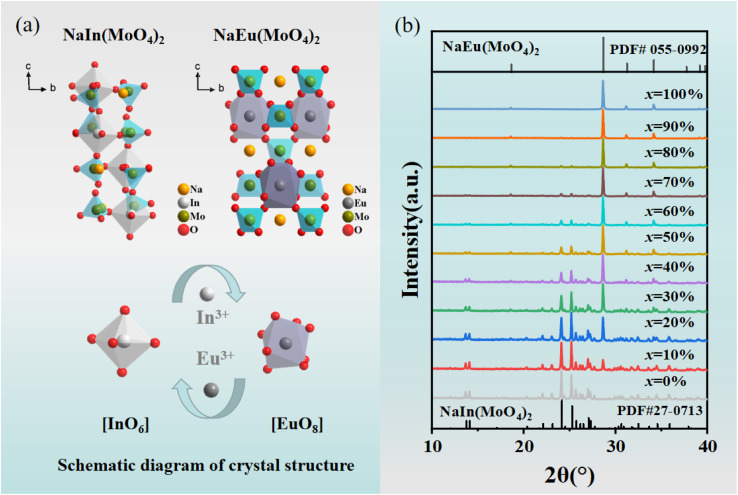
(a) Schematic crystal structures of NIMO and NEMO. (b) XRD patterns of Eu^3+^ doped NIMO samples.

The cell parameters of NIMO can be obtained from the standard structure in the inorganic crystal structure database (ICSD): the space group is *P*1, *a* = 7.29 Å, *b* = 7.29 Å, *c* = 15.06 Å, *α* = 90.01°, *β* = 90.02°, *γ* = 99.11°, *V* = 788.93 Å^3^, *Z* = 4, belonging to the triclinic system.^31^ In^3+^ (CN = 6, *r* = 0.81 Å) is less than Eu^3+^ (CN = 6, *r* = 0.947 Å). The cell parameters for NaEu(MoO_4_)_2_ (NEMO) as follows: the space group is *I*4, *a* = 5.35 Å, *b* = 5.35 Å, *c* = 11.64 Å, *α* = 90°, *β* = 90°, *γ* = 90°, *V* = 332.76 Å^3^, *Z* = 2, belonging to the tetragonal crystal system.^32^ Eu^3+^ (CN = 8, *r* = 1.07 Å) is greater than In^3+^ (CN = 8, *r* = 0.92 Å). From Fig. 1(a), it can be seen that the NIMO unit cell contains six-coordinated [InO_6_] and four coordinated [MoO_4_], while the NEMO unit cell contains eight-coordinated [EuO_8_] and four-coordinated [MoO_4_]. As to NIMO hosts, In^3+^ ions are located in anti-centrosymmetric positions of the unit cell, substitution of Eu^3+^ ions can reduce the symmetry and breaks the parity prohibition, while causes energy absorption and emission. In contrast, In^3+^ ion replaces Eu^3+^ in the octahedral dodecahedron of the NEMO unit cell, not in the anti-symmetric center, while the weaker energy transfer occurs. It is reported that the probability of successful replacement of luminescent centers depends on the degree of difference in ion radius. The formula is as follows:^33^1
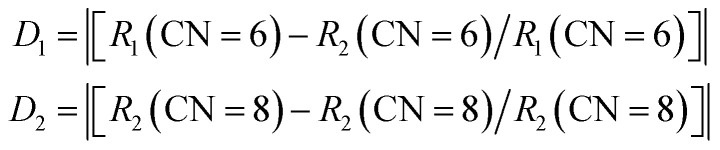
where *R*_1_ represents the ionic radii of In^3+^ and *R*_2_ represents the ionic radii of Eu^3+^. According to formula (1), *D*_1_ = 18.4%, *D*_2_ = 13.7%, are all below 30%, representing the possibility of Eu^3+^ and In^3+^ effectively occupying two different structures. In addition, due to differences in coordination environment, the cell volume of NIMO is 2.37 times that of NEMO, it can be clearly known that when the crystal field undergoes severe distortion and changes, new CET relationship regulation laws will occur in such materials. In Fig. 1(b), it can be clearly seen that when the Eu^3+^ doping concentration is below 20%, the powders mainly exhibit diffraction peaks of NIMO with a PDF card no. 27-0713. However, as the Eu^3+^ doping concentration further increases, diffraction peak of NEMO with a PDF card no. 055-0992 appears and gradually strengthens. When the Eu^3+^ doping concentration exceeds 70%, the main manifestation is the diffraction peak of NEMO. In the field of materials science, research on the structural phase transition caused by the increase of doping components is very interesting.^34^ According to XRD analysis, there is a typical two-phase mixing and gradual transitions with varying Eu^3+^ concentrations in this material, which will become an important origin for regulation of emission properties.

### 3.2 Concentration-dependent luminescence and dynamics properties

**Fig. 2:**
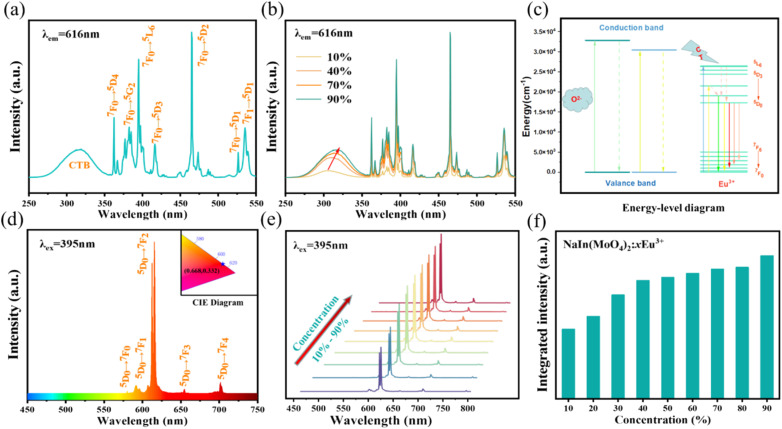
(a) PLE spectrum of NIMO:90%Eu^3+^ sample. (b) PLE spectrum of NIMO:*x*Eu^3+^ with different Eu^3+^ doping concentrations. (c) Energy level diagram of Eu^3+^. (d) PL spectrum of NIMO:90%Eu^3+^ sample, inset is its CIE coordinate. (e) Emission peaks of NIMO:*x*Eu^3+^. (f) Integrated intensity of NIMO:*x*Eu^3+^

Eu^3+^ is one of the most popular red light emitting centers due to its typical ^5^D_0_–^7^F_1_,_2_ transitions. The photo-luminescence excitation (PLE) and emission (PL) spectra of Eu^3+^ doped NIMO are given in Fig. 2. In Fig. 2(a), the PLE spectrum contains a wide charge transfer band (CTB) and a series of narrow band absorption peaks. The broadband absorption peak range 260–360 nm with a central wavelength of 305 nm is attributed to the charge transfer from the 2p orbitals of O^2−^ in MoO_4_^2−^ to the unfilled 4f orbitals in Eu^3+^. The stronger covalent bond between W and O reduces the probability of O^2−^ migrating to Eu^3+^, showing a relatively weak charge absorption band.^35^ In addition, the narrow band absorption peaks range from 360–450 nm are derived from the transitions in 4f orbitals of Eu^3+^. Some strong peaks appear at 362 nm, 382 nm, 395 nm, 416 nm, and 465 nm, corresponding to transitions of ^7^F_0_–^5^D_4_, ^7^F_0_–^5^G_J_/^5^L_7_, ^7^F_0_–^5^L_6_, ^7^F_0_–^5^D_3_ and ^7^F_0_–^5^D_2_, respectively. These wavelengths exactly match the emission wavelengths of near-ultraviolet and blue light chips, making them valuable in red and white LED applications. PLE spectra with different Eu^3+^ doping concentrations are presented in Fig. 2(b). It can be clearly seen that the CTB gradually undergoes a red shift, which is due to a shortening of the bond length between Eu and O as the crystal phase changes, resulting in a decrease of the energy needed to transfer electrons from the 2p orbital of O^2−^ to the 4f orbital of Eu^3+^. This also corresponds to the experimental conclusion of XRD patterns. In contrast, the positions of a series of sharp absorption peaks remain constant due to energy level transitions within the 4f–4f orbitals. For better understanding of charge transfer process and energy level transitions, Fig. 2(c) presents the classical energy level diagram of Eu^3+^ ions with the main excitation and emission processes. In molybdate luminescent materials, when the excited state electron transitions to various energy levels from the ground state, the main emission peaks belong to ^5^D_0_–^7^F_J_, which is due to the large phonon threshold of molybdate matrix and the high non-radiative transition rate between high-energy levels.

In Fig. 2(d), the PL spectra of Eu^3+^ are measured at the excitation wavelength of 395 nm. It can be seen that all spectra exhibit narrowband emissions at 592 nm, 616 nm, 656 nm, and 702 nm, corresponding to transitions ^5^D_0_–^7^F_1_, ^5^D_0_–^7^F_2_, ^5^D_0_–^7^F_3_, and ^5^D_0_–^7^F_4_. It can be clearly seen that the electric dipole transition ^5^D_0_–^7^F_2_ is dominant and gradually enhances with increased Eu^3+^ concentration. The previous report have shown that the ratio of ^5^D_0_–^7^F_2_ to ^5^D_0_–^7^F_1_ not only determines the color of the emitted light but also reflects the local symmetry of Eu^3+^^26^. The calculation results in Fig. S1† indicates the ratios remain unchanged, which suggests that the increasing Eu^3+^ doping concentration does not alter the low inversion symmetry of Eu^3+^ from the octahedral and dodecahedral substitution and maintains stable color purity simultaneously.^36,37^ In the inset of Fig. 2(d), the CIE coordinates of NIMO:90%Eu^3+^ is (*x*, *y*) = (0.668, 0.332), similar to the internationally recognized standard for red color (0.666, 0.334). Moreover, the color purity of all samples is higher than 96% by calculation.^38^ In Fig. 2(e) and (f), the emission peaks and integral intensity of NIMO:*x*Eu^3+^ with Eu^3+^ doping concentrations from 10% to 90% are measured and calculated. It is worth noting that the integral intensity of samples gradually increase with the enhanced Eu^3+^ doping concentrations, and no traditional concentration quenching phenomenon is observed.

**Fig. 3:**
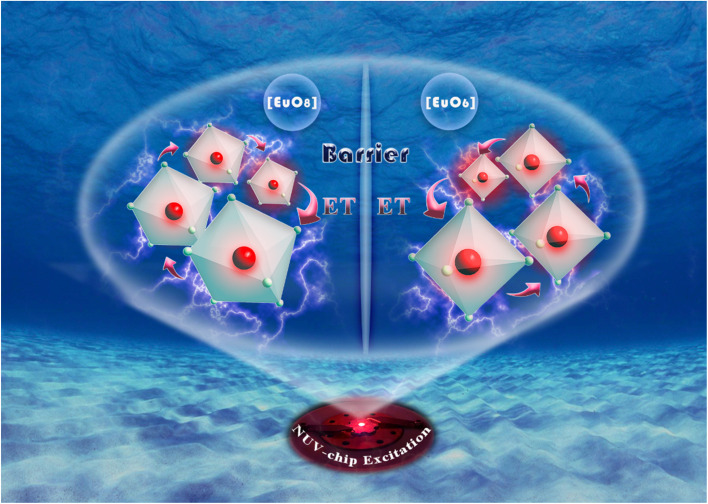
Schematic diagram of the energy transfer relationship in the NIMO:*x*Eu^3+^.

In traditional luminescent materials, emitters can homogeneously disperse into the interior host matrix by replacing the equivalent ions that have similar ionic radii, the occurrence of concentration quenching is mainly due to the accelerated CET with closer distance between dopants.^39^ However, a different mode of energy transfer relationship on concentration quenching inhibition should be highlighted in NIMO:*x*Eu^3+^, and the energy transfer relationship model is represented in Fig. 3. It can be clearly seen that, as the Eu^3+^ concentration increases, the second phase of NEMO gradually generates and manifolds. There exist three pathways for CET between emitters in such materials, namely Eu–Eu in the six-coordinated structure, Eu–Eu in the eight-coordinated structure, and Eu–Eu between the six-coordinated and eight-coordinated structures. According to Blasse's report, when the distance between emitters is less than 5 Å, energy transfer is prone to occur between them.^40^ On the contrary, when the distance is greater than 5 Å, energy transfer is less effective. In NIMO, if In^3+^ is completely occupied by Eu^3+^, the closest distance between emission centers is 5.8827 Å. But in NEMO, the closest distance between emission centers is 5.3470 Å. Therefore, the sustained fluorescence enhancement in NIMO:*x*Eu^3+^ indicates that the average distance between Eu^3+^ emitters is increasing. It should be emphasized that the main contribution to increasing the average spacing comes from the increase in lattice interface barriers between six-coordinated Eu unit and eight-coordinated Eu unit. The increasing interface barriers can effectively block the CET between the two-phase structures and mount up the number of emitted photons for enhancing emission.^41,42^ In addition, it is observed that the emission intensity of NIMO:90%Eu^3+^ is about 1.3 times that of NEMO in Fig. S2,† indicating an efficient red light emission can be achieved under this CET mechanism. Moreover, the absolute quantum yield of the NIMO:90%Eu^3+^ phosphor is measured in Fig. S3† and a remarkable value of 90.87% is obtained under the excitation wavelength of 395 nm.

**Fig. 4:**
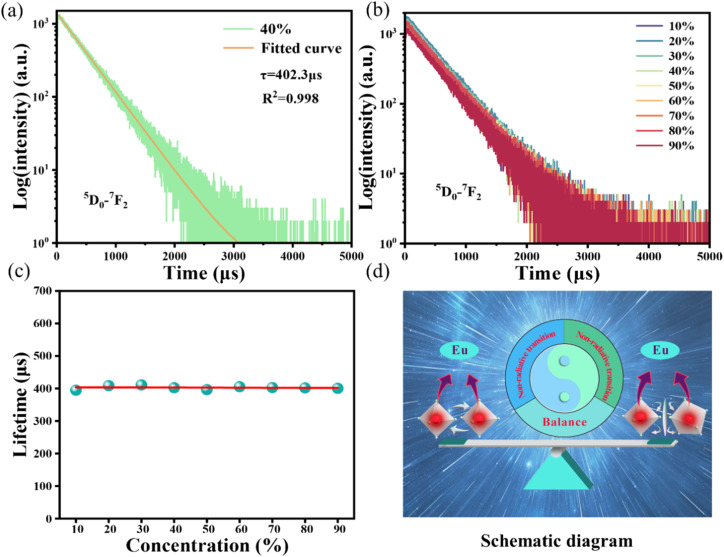
(a) Fluorescence decay curve of NIMO:40%Eu^3+^. (b) Fluorescence decay curves of NIMO:*x*%Eu^3+^. (c) Fluorescence decay time constants of NIMO:*x*%Eu^3+^. (d) Schematic diagram of non-radiative transition regulation mechanism in NIMO:*x*%Eu^3+^.

In Fig. 4, the fluorescence lifetime curves are measured to investigate the microscopic population process of Eu^3+^ activators in NIMO:*x*Eu^3+^ phosphors. Fig. 4(a) displays the fluorescence decay curve of NIMO:40%Eu^3+^ in ^5^D_0_–^7^F_2_ transition, which can be well fitted by the following first-order exponential equation:^43^

2 *I*(*t*) = *A* exp(−*t*/*τ*)

where *I*(*t*) represents the luminous intensity at time *t*, *A* is a constant, and *τ* is the average single photon fluorescence lifetime. The fitting result shows that the decay time constant is 402.3 μs. In Fig. 4(b) and (c), it is interesting to find that all lifetimes of Eu^3+^ doping concentrations ranging from 10% to 90% are almost unchanged and around 400 μs, this is also means that the total electron transition rates do not change with the variation of Eu^3+^ doping concentrations. As is known that, the electron transition rate includes two parts: radiative transition rate and non-radiative transition rate. Generally, when the environment of the luminescent center is stable, the radiation transition rate remains almost constant, but the non-radiative transition rate is related to the change in crystal field. In NIMO:*x*Eu^3+^, there are two main factors that affect non-radiative transition, one is the CET between Eu ions in the six-coordinated structure, the other is the CET between Eu in the six-coordinated structure and Eu in the eight-coordinate structure. In the six-coordinated structure of NIMO, the increasing Eu^3+^ doping concentration can shorten the distance between them, while increasing the non-radiative transition rate. Complementarily, the lattice interface barriers related to Eu in the six-coordinated structure and in the eight-coordinated structure will also increase with the increasing Eu emitters, which can decrease the non-radiative transition rate by blocking the CET between them. The unchanged lifetimes in NIMO:*x*Eu^3+^ indicate that the increasing non-radiative transition rate is equivalent to the decreasing value, and cancels out each other. To better understand the regulatory mechanism of non-radiative transition rate, a vivid schematic diagram is shown in Fig. 4(d). Furthermore, the fluorescence lifetime of NEMO is measured and calculated as 336 μs in Fig. S4,† indicating the serious non-radiative transition processes among their Eu emitters.

### 3.3 Temperature-dependent emission spectra and dynamics

**Fig. 5:**
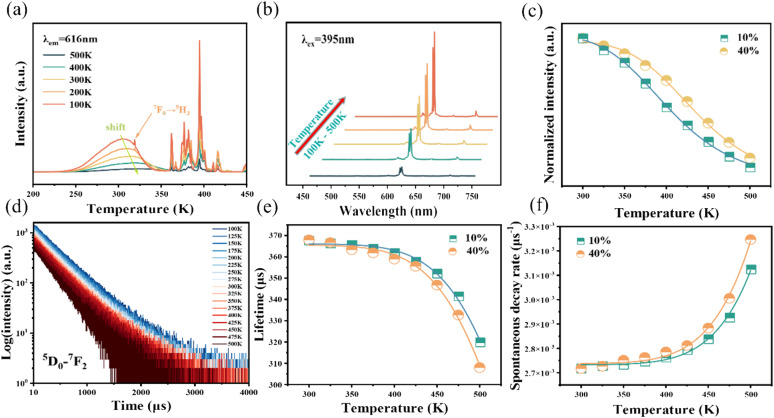
(a) Temperature-dependent PLE spectra of NIMO:40%Eu^3+^. (b) Temperature-dependent PL spectra of NIMO:40%Eu^3+^. (c) Temperature dependence of normalized integral intensities of NIMO:10%Eu^3+^ and NIMO:40%Eu^3+^. (d) Temperature-dependent fluorescence decay curves of NIMO:40%Eu^3+^. (e) Temperature-dependent fluorescence decay time constants of NIMO:10%Eu^3+^ and NIMO:40%Eu^3+^. (f) Temperature-dependent spontaneous decay rates of NIMO:10%Eu^3+^ and NIMO:40%Eu^3+^.

As is well known, there is a strong dependence of luminescence intensity on environmental temperature, which is known as thermal-quenching effect. To reveal the thermal-quenching properties in the two-phase mixing NIMO:*x*Eu^3+^ materials, the PLE and PL spectra of NIMO:*x*Eu^3+^ (*x* = 10% and 40%) with the varying temperature range from 100 K to 500 K are displayed in Fig. 5(a) and (b). In Fig. 5(a), it can be seen that absorption bands of matrix in NIMO:40%Eu^3+^ have a red shift with the increasing temperature, which is due to the different sensitivity to temperature for the overlapping PLE spectra in the two-phase structures. In Fig. 5(b), it is evident that the PL intensity of NIMO:40%Eu^3+^ gradually decreases with increasing temperature, but the position and proportion of the emission peaks remain almost unchanged, indicating a good color thermal stability. As the PLE and PL spectra measured in Fig. S5,† if the luminous intensity at 300 K is used as a reference, when temperature rises to 400 K, the emission intensities of NIMO:10%Eu^3+^ and NIMO:40%Eu^3+^ remain at 53.5% and 76.3%, which indicates that sample with high Eu^3+^ doping concentration exhibits good luminescence stability. In Fig. 5(c), the activation energy is obtained by the following formula:^44^3
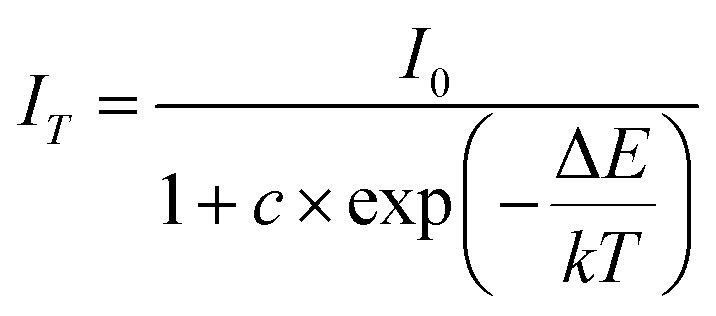
where *I*_0_ represents the initial luminous intensity, Δ*E* is the thermal activation energy, *k* is the Boltzmann constant, *T* is the real-time temperature, and *c* is the constant. By fitting, the thermal activation energy of NIMO:10%Eu^3+^ and NIMO:40%Eu^3+^ are 0.327 eV and 0.388 eV. It can be seen that samples with higher doping concentrations have higher thermal activation energy, thus exhibiting good luminescence stability. It should be noted that the CET mainly occurs among the six-coordinated Eu^3+^ ions in NIMO:10%Eu^3+^ phosphor. But in NIMO:40%Eu^3+^, the gradually increasing lattice interface barriers can effectively block the CET, thereby inhibiting the thermal activation process of materials.

To further reveal the temperature quenching of the emission intensity in such materials, the dynamics of the ^5^D_0_–^7^F_J_ transition in NIMO:10%Eu^3+^ and NIMO:40%Eu^3+^ as a function of temperature are determined and shown in Fig. 5(d), which shows a regular change with increasing temperature. Based on the multi-phonon-relaxation theory, the total spontaneous emission rate of ^5^D_0_ can be written as follows:^45^

4 *W*_T_ = *W*_R_ + *W*_NR_(0) × (1 − exp(ℏ*ω*/*kT*))^−Δ*E*/ℏ*ω*^

*W*_T_ represents the total spontaneous emission rate, *W*_R_ represents the radiative transition rate, and *W*_NR_(0) represents the non-radiative transition rate at 0 K. By fitting, it is deduced that *W*_R_ = 0.00273 ms^−1^ and *W*_NR_(0) = 41.5 ms^−1^ for NIMO:10% Eu^3+^, *W*_R_ = 0.00274 ms^−1^ and *W*_NR_(0) = 31.4 ms^−1^ for NIMO:40%Eu^3+^. It can be seen that *W*_R_ remain constant between NIMO:10%Eu^3+^ and NIMO:40%Eu^3+^, however, *W*_NR_(0) reduces by approximately 25% with increasing Eu^3+^ doping concentrations. This experiment results further indicate that the increase in the average spacing of Eu^3+^ by lattice interface barriers can effectively suppress non-radiative energy loss, as described in the above section.

### 3.4 Applications in red and warm white LEDs

**Fig. 6:**
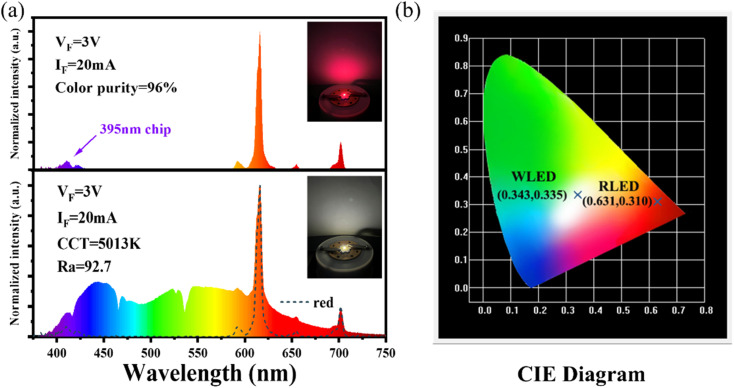
(a) Electroluminescence performance of the fabricated red and white LED devices, the insets show their working conditions. (b) Corresponding chromaticity coordinates in the CIE diagram.

The luminescence intensity of Eu^3+^ doped red phosphors is crucial for supplementing the red light component in commercial fluorescent powder. A red-emitting LED device is packaged by combining a 395 nm near-UV chip and the NIMO:90%Eu^3+^ phosphors in Fig. 6(a). The device emits bright red light visible to the naked eye (inset of Fig. 6(a)) and shows high color purity of 96%, representing its potential application in improving the color rendering performance of lighting devices. To confirm its practical application in warm white indoor lighting, a white LED device is encapsulated by combining the commercial BAM:Eu^2+^ blue phosphors, YAG:Ce^3+^ yellow phosphors and the as-prepared NIMO:90%Eu^3+^ onto the 395 nm near-UV chip in Fig. 6(a). As wishes, the coated white LED displays a bright white light emitting with slightly yellow (the lower part of Fig. 6(a), inset). The fabricated white LED shows an effective lighting performance with CIE chromaticity coordinates of (0.343, 0.335) (Fig. 6(b)), CRI of 92.1, CCT of 5013 K, color realism of 86.9, and color saturation of 101.1. All the experimental results indicates that the two-phase mixing NIMO:*x*Eu^3+^ materials have potential application prospects in warm white LEDs.

## 4. Conclusions

In summary, a series of highly efficient NIMO:*x*Eu^3+^ (*x* = 10–90%) red light phosphors are successfully synthesized using a high-temperature solid-state method and concentration quenching inhibition is found in this system. The crystal structure, PLE and PL spectra, fluorescent dynamics and temperature-dependent luminescence properties are performed to comprehensively investigate its concentration quenching mechanism. As the Eu^3+^ doping concentration increases, the coexistence of Eu six-coordinated and eight-coordinated phases appears in the as-prepared materials. The increase lattice interface barriers can enlarge the average spacing of Eu in material, effectively suppress their CET, and enhance the red emission. The fluorescence lifetime does not change with the of Eu^3+^ doping concentration, indicating that the increase of CET in Eu six-coordination units and the decrease of CET by lattice interfaces barriers are mutually constrained. Temperature-dependent luminescence performance analysis shows that due to the regulatory effect of lattice interface barriers, the NIMO:40%Eu^3+^ sample has better luminescence thermal stability, and the non-radiative transition rate is suppressed by 25% compared to the NIMO:10%Eu^3+^. Finally, the encapsulated device displays an effective warm white light performance with suitable CIE coordinates of (0.343, 0.335), low CCT of 5013 K, and high CRI of 92.7. In brief, the experimental results show a new mechanism for regulating CET relationships in the two-phase mixing materials, and lays an important foundation for exploration and preparation of new and efficient luminescent materials for warm white LEDs.

## Conflicts of interest

There are no conflicts to declare.

## Supplementary Material

RA-013-D3RA05873E-s001
