# Supplementary material for: Concentration quenching inhibition and fluorescence enhancement in Eu3+-doped molybdate red phosphors with two-phase mixing
Source: RSC Adv. 2023 Nov 1;13(44):31167–75. doi: 10.1039/d3ra05873e (PMC10619205; doi:10.1039/d3ra05873e)
Supplement: RA-013-D3RA05873E-s001 [file RA-013-D3RA05873E-s001.pdf]

Electronic Supplementary Material (ESI) for RSC Advances.
This journal is © The Royal Society of Chemistry 2023

Supplementary Information

Concentration Quenching Inhibition and Fluorescence Enhancement in Eu^{3+}-doped Molybdate Red Phosphors with Two-phase Mixing

Shuanglai Liu [ab],Yimin Yan [b], Xiaohan Liu [b], Zheqian Cui [b], Shiheng Jia [b], Yiwen Xing [b], Shuang Guo [b], Bao Wang [b], Yunfeng Wang [b]*

[a] *School of Physics and Electronic Engineering, Zhengzhou University of Light Industry, Zhengzhou* 450000*, P. R. China.*

[b] *School of Information Engineering, Nanyang Institute of Technology, Nanyang* 473004*, P. R. China.*

* Corresponding author. wangyunfeng@*nyist.edu.cn*

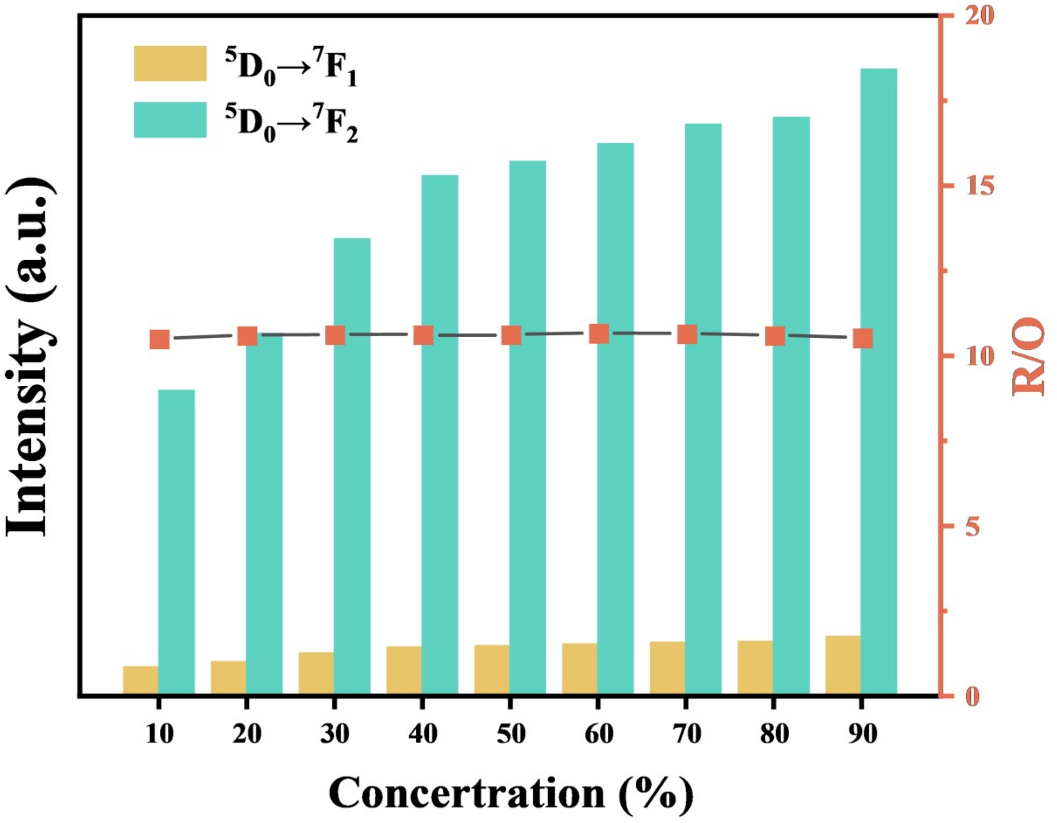

Fig. S1. Integrated emission intensity of NIMO:*x*Eu^{3+} and the ratios of $^5D_0$-$^7F_2$ /$^5D_0$-$^7F_1$ (R/O) with different Eu^{3+} doping concentrations.

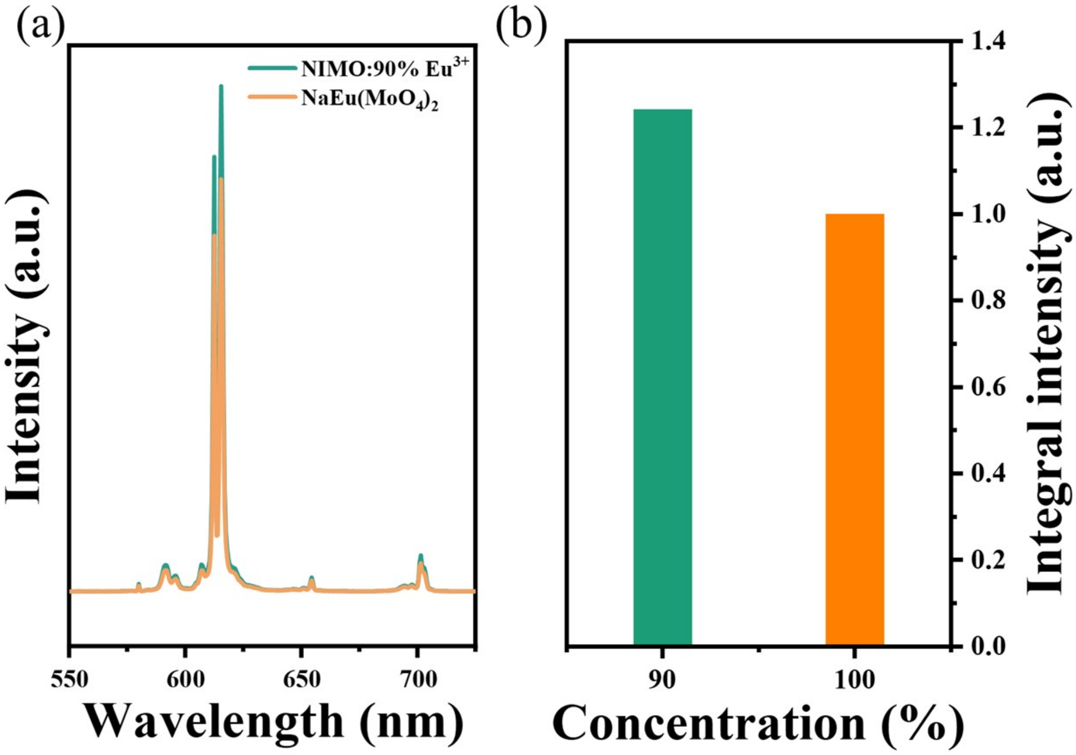

Fig. S2. (a) Comparison of PL spectra between NIMO:90%Eu^{3+} and NEMO. (b) integrated PL intensity of NIMO:90%Eu^{3+} and NEMO.

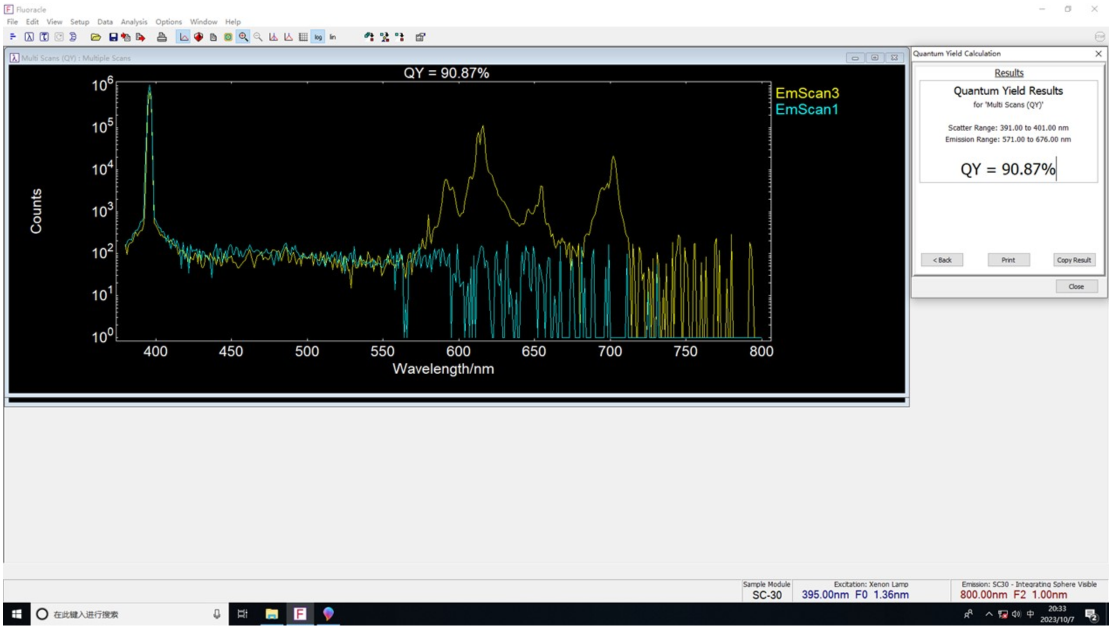

Fig. S3. Absolute quantum yield of NIMO:90%Eu^{3+} phosphor with the excitation wavelength of 395nm

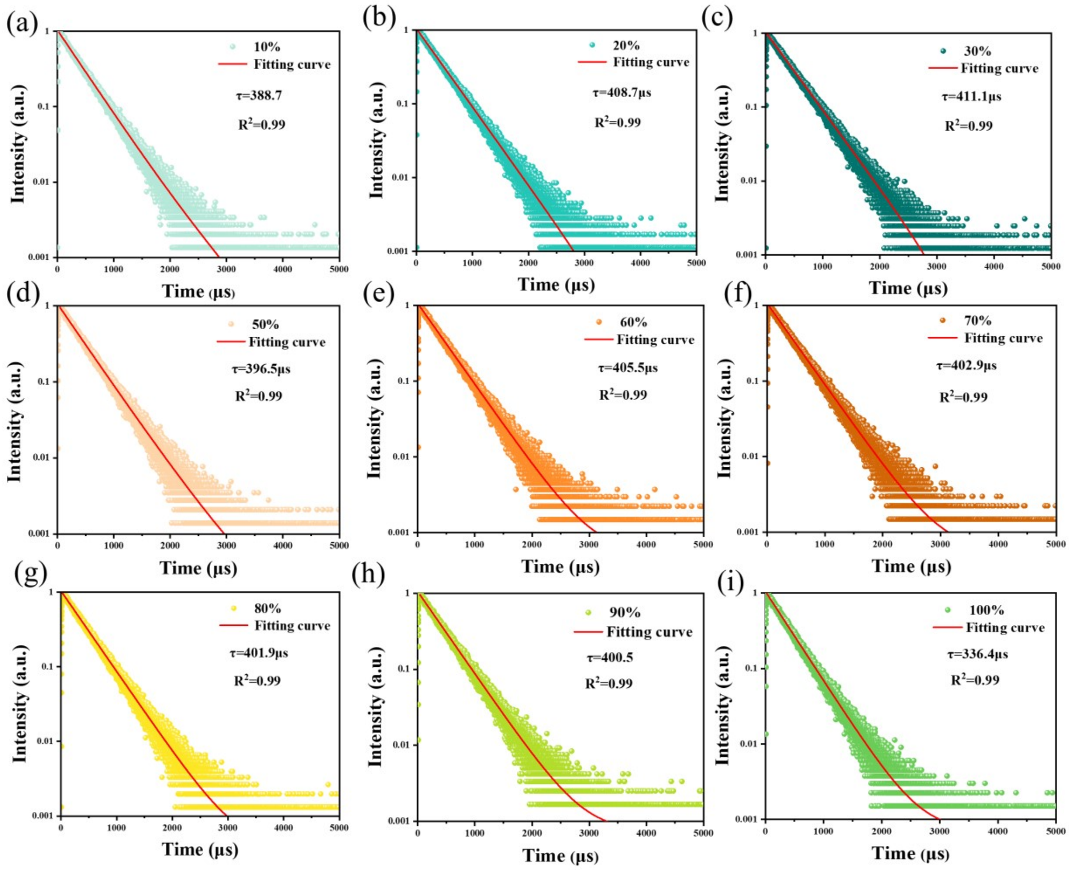

Fig. S4. Fluorescence decay curves and fitting lines of $NaIn(MoO_4)_2$:*x*%Eu^{3+} with different Eu^{3+} doping concentrations.

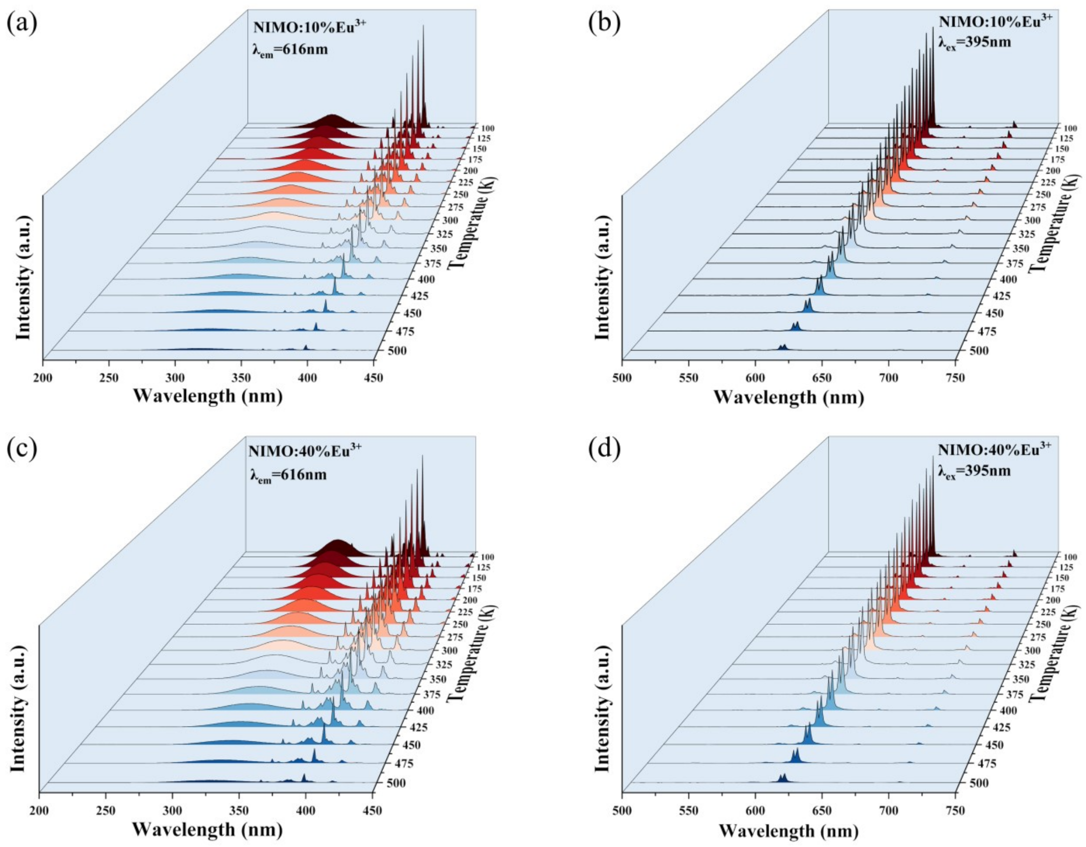

Fig. S5. (a) PL and (b) PLE spectra of $NaIn(MoO_4)_2$:10%Eu^{3+} with varying temperature ranging from 100 to 500K. (c) PL and (d) PLE spectra of $NaIn(MoO_4)_2$:40%Eu^{3+} with varying temperature ranging from 100 to 500K.
